# Successful resolution of upper jejunal obstruction caused by bezoars using three sessions of gastroscopic lithotripsy

**DOI:** 10.1055/a-2677-9805

**Published:** 2025-08-22

**Authors:** Wanling Li, Bingqiang Zhang

**Affiliations:** 1 568864 Gastroenterology, University-Town Hospital of Chongqing Medical University, Chongqing, China; 2 Chongqing Hospital of Jiangsu Province Hospital, Chongqing, China; 3 623680 The Peopleʼs Hospital of Qijiang District Chongqing, Chongqing, China

A 57-year-old woman presented with upper abdominal pain for 2 months and vomiting for 10 days. Computed tomography (CT) scan revealed an upper jejunal obstruction caused by multiple bezoars measuring approximately 40 × 42 mm ( Fig. 1 ). Gastroscopy performed 2 months earlier had identified a 7 × 4-cm bezoar in the stomach ( Video 1 ). No intervention was performed at that time, and the patient was prescribed a proton pump inhibitor. Her medical history included laparoscopic cholecystectomy 4 years prior.

**Fig. 1:**
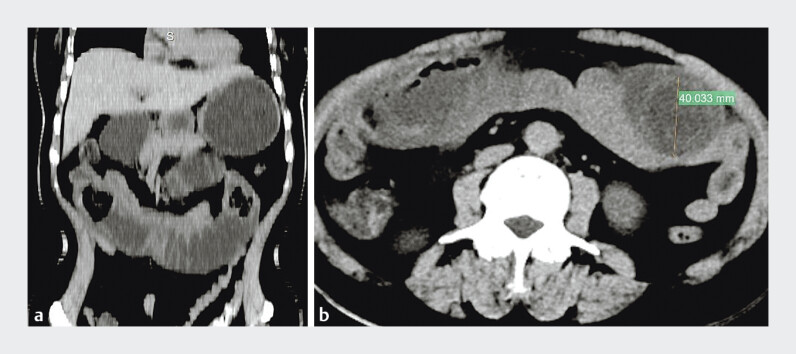
Coronal ( **a** ) and axial ( **b** ) computed tomography images showing upper jejunal obstruction caused by numerous bezoars, measuring approximately 40 × 42 mm.

A 7 × 4-cm gastric bezoar identified 2 months earlier in a 57-year-old woman had migrated to the horizontal segment of the duodenum and upper jejunum. Attempts to remove the bezoars using a snare were unsuccessful. Subsequently, the bezoars were successfully removed using three sessions of gastroscopic lithotripsy.Video 1

During the first gastroscopy, a large bezoar was visualized in the descending segment of the duodenum. After it was removed using a snare, another large bezoar was identified in the horizontal segment of the duodenum, which could not be fragmented using the snare ( Fig. 2 ). A second gastroscopy successfully removed the bezoar from the horizontal segment, but another bezoar was detected in the upper jejunum, which also could not be fragmented ( Fig. 3 , Video 1 ). Third time of gastroscopy, we repeatedly destroyed the bozoars with the snare, the obstruction was relieved ( Fig. 4 ).

**Fig. 2:**
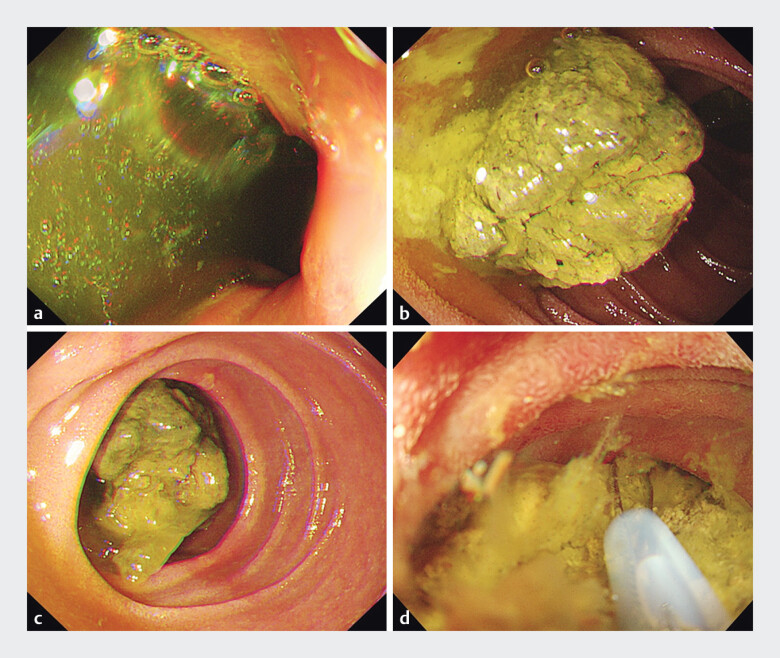
Endoscopic findings during the first gastroscopy. **a** A large bezoar identified in the descending segment of the duodenum. **b** Close-up view of the bezoar. **c** Another large bezoar identified in the horizontal segment of the duodenum. **d** Attempted fragmentation of the horizontal duodenal bezoar using a snare, which was unsuccessful.

**Fig. 3:**
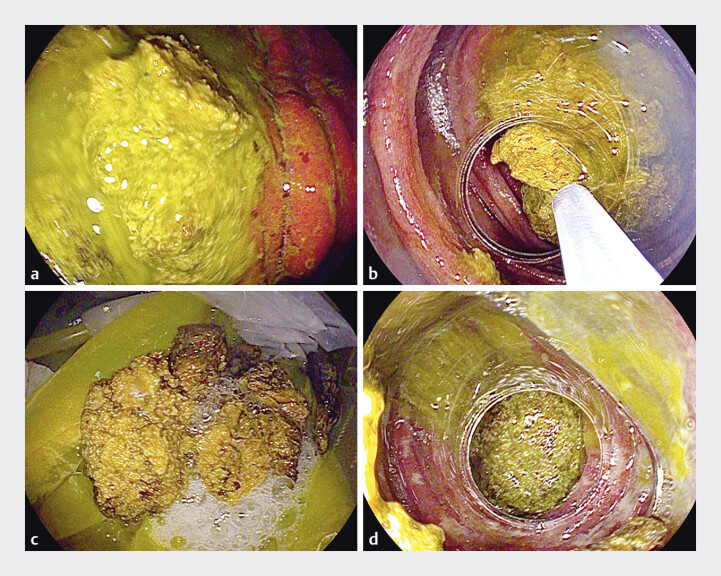
Endoscopic findings during the second gastroscopy. **a** Bezoars seen in the horizontal segment of the duodenum. **b** Attempted removal of the duodenal bezoars. **c** Duodenal bezoars were successfully removed. **d** Additional bezoars were found in the upper jejunum, which could not be fragmented.

**Fig. 4:**
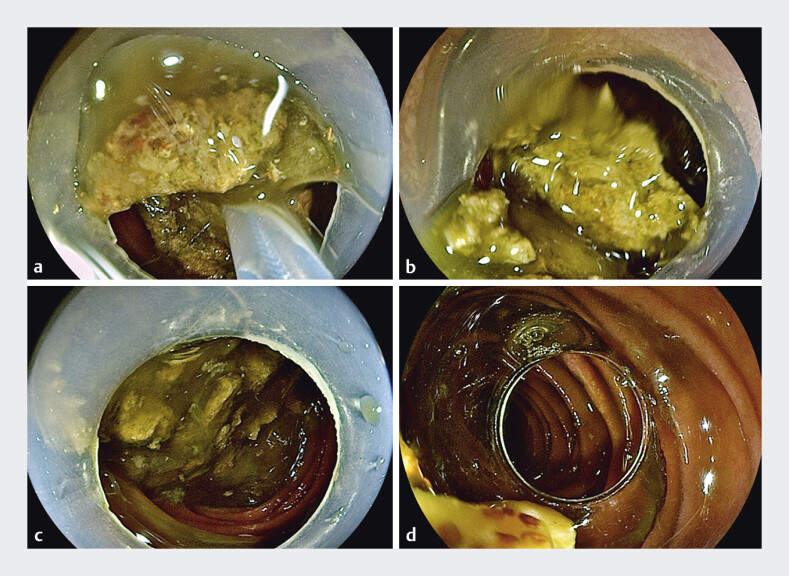
Endoscopic findings during the third gastroscopy. **a** Multiple bezoars were visualized. **b** The bezoars were repeatedly fragmented using the snare. **c** The bezoars were successfully fragmented. **d** The obstruction caused by bezoars was relieved.

A follow-up CT scan confirmed complete resolution of the jejunal obstruction ( Fig. 5 ). The patient resumed a normal diet and reported no further symptoms during telephone follow-up.

**Fig. 5:**
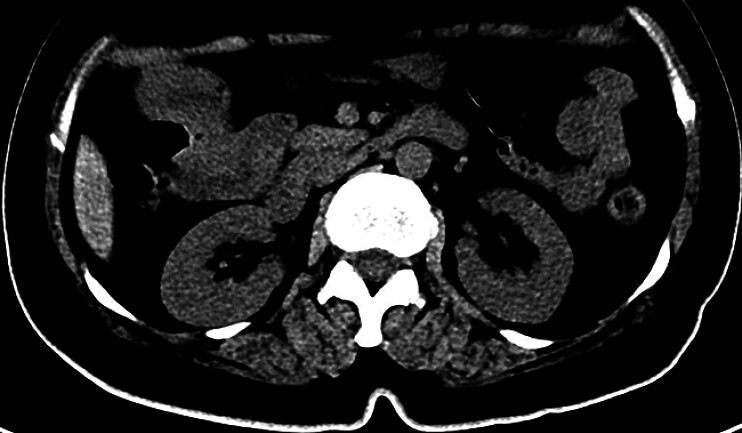
Follow-up computed tomography scan did not show any signs of jejunal obstruction.

The most common type of bezoars is plant-based, typically observed in older patients with impaired gastric motility and high consumption of black jujube 1 . Plant-based bezoars in the stomach can often be fragmented using a snare 2 . However, gastrotomy or laparoscopic surgery is recommended for bezoars with a large diameter and hard shell. Furthermore, intestinal obstructions caused by bezoars usually require surgical intervention 3 . In this case, the jejunal obstruction caused by bezoars was successfully treated using three sessions of endoscopic lithotripsy, representing the first reported case of successful resolution of upper jejunal obstruction using gastroscopic lithotripsy.

In this case, bezoars obstructed the jejenum and caused mucosal ulceration. This case presented a significant challenge for gastroscopic lithotripsy due to procedural complexity, risks, and time requirements. Careful, staged fragmentation of the bezoar core was crucial to achieving success. With advances in endoscopic techniques, particularly the introduction of laser lithotripsy, endoscopic management of bezoars is expected to become increasingly effective 4 . In the future, small intestinal obstruction caused by bezoars may also be treatable using enteroscopy.

Endoscopy_UCTN_Code_TTT_1AP_2AD

## Notes

Endoscopy E-Videos https://eref.thieme.de/e-videos
*E-Videos* is an open access online section of the journal *Endoscopy* , reporting on interesting cases and new techniques in gastroenterological endoscopy. All papers include a high-quality video and are published with a Creative Commons CC-BY license. Endoscopy E-Videos qualify for HINARI discounts and waivers and eligibility is automatically checked during the submission process. We grant 100% waivers to articles whose corresponding authors are based in Group A countries and 50% waivers to those who are based in Group B countries as classified by Research4Life (see: https://www.research4life.org/access/eligibility/ ). This section has its own submission website at https://mc.manuscriptcentral.com/e-videos .
